# Endolysins against Streptococci as an antibiotic alternative

**DOI:** 10.3389/fmicb.2022.935145

**Published:** 2022-08-02

**Authors:** Kuan Yee Wong, Megat Hamzah Megat Mazhar Khair, Adelene Ai-Lian Song, Mas Jaffri Masarudin, Chou Min Chong, Lionel Lian Aun In, Michelle Yee Mun Teo

**Affiliations:** ^1^Department of Biotechnology, Faculty of Applied Sciences, UCSI University, Kuala Lumpur, Malaysia; ^2^Department of Microbiology, Faculty of Biotechnology and Biomolecular Sciences, Universiti Putra Malaysia, Selangor, Malaysia; ^3^Department of Cell and Molecular Biology, Faculty of Biotechnology and Biomolecular Sciences, Universiti Putra Malaysia, Selangor, Malaysia; ^4^Department of Aquaculture, Faculty of Agriculture, Universiti Putra Malaysia, Selangor, Malaysia

**Keywords:** endolysins, streptococci, phages, enzybiotic, antibiotic alternative

## Abstract

Multi-drug resistance has called for a race to uncover alternatives to existing antibiotics. Phage therapy is one of the explored alternatives, including the use of endolysins, which are phage-encoded peptidoglycan hydrolases responsible for bacterial lysis. Endolysins have been extensively researched in different fields, including medicine, food, and agricultural applications. While the target specificity of various endolysins varies greatly between species, this current review focuses specifically on streptococcal endolysins. *Streptococcus* spp. causes numerous infections, from the common strep throat to much more serious life-threatening infections such as pneumonia and meningitis. It is reported as a major crisis in various industries, causing systemic infections associated with high mortality and morbidity, as well as economic losses, especially in the agricultural industry. This review highlights the types of catalytic and cell wall-binding domains found in streptococcal endolysins and gives a comprehensive account of the lytic ability of both native and engineered streptococcal endolysins studied thus far, as well as its potential application across different industries. Finally, it gives an overview of the advantages and limitations of these enzyme-based antibiotics, which has caused the term enzybiotics to be conferred to it.

## Introduction

The unfortunate development of antibiotic-resistant strains is a natural phenomenon and is further exacerbated following the misuse and overuse of antibiotics, especially in the medical and agricultural industries (Roach and Donovan, 2015). As a result, antimicrobial resistant (AMR) pathogen-associated hospital-acquired infections caused by AMR strains have killed 99,000 people annually in the United States and are expected to kill 10 million people worldwide by 2050 (Aslam et al., 2018). The release of non-metabolized antibiotics or residues into the environment through feces can also accelerate the emergence of multidrug resistance (MDR) and AMR bacterial infections in society (Aslam et al., 2018; Alves-Barroco et al., 2020). Many bacterial infections are no longer treatable by common antibiotics (Prestinaci et al., 2015). In recent years, outbreaks of AMR bacterial infections, including *Streptococcus* spp., toward multiple antibiotics have been reported as a major crisis not only in the healthcare sector but also in agriculture and aquaculture industries.

*Streptococcus* spp. are Gram-positive, facultative anaerobes, non-motile, non-spore-forming, and oxidase-negative with either α-, β-, or non-hemolytic (γ) disposition (Pradeep et al., 2016; Renzhammer et al., 2020). *Streptococcus* spp. are classified by the expression of different Lancefield antigens found on the cell wall. Lancefield grouping is the traditional method which subdivides *Streptococcus* genus into 20 groups based on the presence of polysaccharides and teichoic acid antigens on the cell wall (Slater, 2007; Efstratiou et al., 2017). Among the groups, both group A *Streptococcus* (GAS) and group B *Streptococcus* (GBS) are the most prevalent human pathogens that cause systemic infections associated with high mortality and morbidity (van Hensbergen et al., 2018). *Streptococcus pyogenes*, which is a GAS bacterium, infects at least 700 million individuals each year, with a mortality rate of 15–30%, resulting in meningitis, toxic shock syndrome, and pharyngitis (Zorzoli et al., 2019). *Streptococcus agalactiae*, a GBS bacterium which is found in the human intestinal and urogenital tract microbiota, is frequently associated with post-cesarean surgical wound infection in pregnant women and infant meningitis (Haenni et al., 2018; Bobadilla et al., 2021). *Streptococcus pneumoniae*, on the other hand, is the major cause of meningitis and many different pulmonary infections, including sepsis (Jiang et al., 2018; Weiser et al., 2018). In the aquaculture industry, *Streptococcus iniae* and *S. agalactiae* have been identified as the most common pathogen associated with streptococcosis outbreaks, resulting in substantial economic losses (Pradeep et al., 2016; Osman et al., 2017). *Streptococcus dysgalactiae* subspecies *equisimilis* and *Streptococcus suis* infections in weaned piglets commonly cause pneumonia or meningitis, thus resulting in increased mortality rates and significant financial losses (Renzhammer et al., 2020).

To reduce the emergence of AMR bacterial strains, phage therapy has been extensively researched as an alternative treatment to bacterial infections. Phage therapy, also known as bacteriophage therapy, was once used to treat bacterial infections in the early 20th century (Duzgunes et al., 2021). However, the popularity of phage therapy was quickly shadowed by the discovery and development of penicillin and other broad-spectrum antibiotics (Murray et al., 2021). Following the discovery of penicillin-resistant *Staphylococcus aureus* in the 1950s and methicillin-resistant *S. aureus* (MRSA) in the 1960s, researchers started looking for antimicrobial alternatives (Guo et al., 2020). Due to the rise of AMR, phage therapy, which includes the use of phage lytic proteins such as endolysins, has become a popular research area as a viable antibiotic alternative. Endolysins or lysins were first described in the lysates of *S. aureus* in the early 1960s (Fischetti, 2018). They are phage-encoded peptidoglycan hydrolases responsible for degrading the peptidoglycan cell wall to release new phage progenies at the end of a phage lytic cycle (Roach and Donovan, 2015). Degradation of the peptidoglycan cell wall disrupts the osmotic balance between cellular and environmental osmotic pressure, resulting in cell lysis and death (Roach and Donovan, 2015; Murray et al., 2021). Due to its high specificity in targeting specific bonds in the peptidoglycan cell wall, endolysins have also been extensively used in various fields, for example, as a natural antibacterial agent in food preservation (Drulis-Kawa et al., 2015; Chang, 2020), as a protein-based narrow-spectrum disinfectant (Hoopes et al., 2009), and a topical skin application that targets MRSA (Totté et al., 2017).

At present, researchers have identified and tested many endolysins to establish their potential as new antimicrobial alternatives. However, no clinical trials involving endolysins targeting *Streptococcus* spp. have been conducted due to the lack of *in vivo* studies (Abdelkader et al., 2019; Abdelrahman et al., 2021). This review aims to discuss the different types of endolysins that target *Streptococcus* spp. and their current applications in different industries.

## Endolysin structure

**FIGURE 1:**
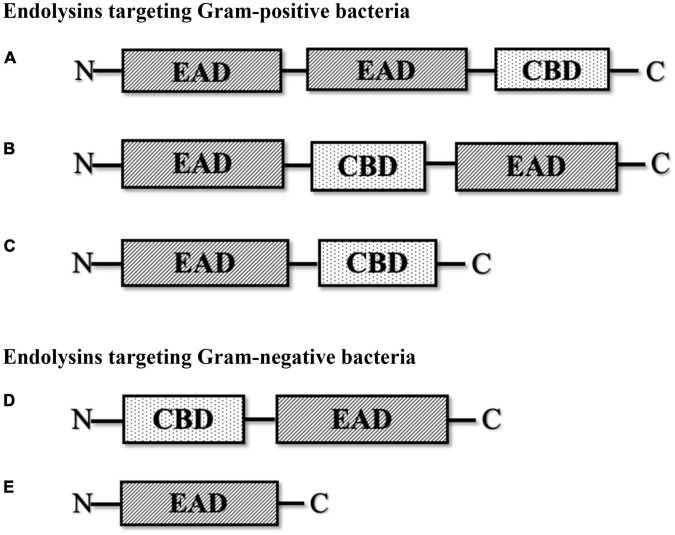
Schematic structure of various types of phage-encoded peptidoglycan hydrolases (endolysin). **(A)** Multi-domain endolysin with more than one enzymatically active domain (EAD) and one C-terminal cell wall-binding domain (CBD). **(B)** Multi-domain endolysin with a centrally located CBD separating two EADs, found in *Streptococcus* phage (λSa2). **(C)** Endolysin with an N-terminal EAD and a C-terminal CBD, another common structure of endolysin, mostly found in streptococcal endolysins and phages infecting *Listeria*, *Clostridium*, and *Bacillus*. **(D)** Endolysin with an N-terminal CBD and a C-terminal EAD, found in some *Pseudomonas* phages (KZ114 and EL188). **(E)** Globular endolysin with only one EAD, found in most Gram-negative phages.

Endolysins are hydrolytic enzymes that can specifically identify and cleave the bacterial peptidoglycan cell wall. Most endolysins comprise a single polypeptide chain, usually greater than 25 kDa, and are composed of an N-terminal enzymatically active domain (EAD) and a C-terminal cell wall-binding domain (CBD), which are linked by a short flexible linker (Figure 1; Zhou et al., 2020). Endolysins are produced and expressed at the end of the phage lytic cycle, releasing phage progeny following degradation of the bacterial peptidoglycan cell wall (Chang, 2020; Zhou et al., 2020). Most endolysins pass through the cell wall with the help of holin proteins, which are small hydrophobic hole-forming proteins. Holin proteins trigger an opening on the cytoplasmic side and allow endolysins to hydrolyze the cell wall (Lin et al., 2017; Abdelrahman et al., 2021; Murray et al., 2021). However, some endolysins possess N-terminal signal peptides and are secreted without the help of holins, while the filamentous phage releases new progenies with an entirely different mechanism that does not lyse or kill the host cells (Schmelcher et al., 2012). The bacterial cell wall, which is made up of chains of N-acetylmuramic acid (MurNAc) and N-acetyl-D-glucosamine (GlcNAc) linked by glycosidic bonds (Vazquez et al., 2018; Dörr et al., 2019), regulates osmotic pressure and prevents the undesired passage of molecules (Dik et al., 2017; Dörr et al., 2019). The peptidoglycan cell wall of Gram-positive bacteria is multilayered, while that of Gram-negative bacteria is mono- or bilayered (Dik et al., 2017) and additionally possesses an outer membrane. Thus, in Gram-positive cells, endolysins can lyse peptidoglycan from the outside of the cell if applied externally, a phenomenon termed “lysis-from-without” as opposed to “lysis-from-within” (Schmelcher et al., 2012). The outer membrane of Gram-negative bacteria usually prevents endolysins from lysis-from-without, unless the endolysin has been engineered with outer membrane-permeabilizing peptides, such as in the case of artilysins (Schmelcher et al., 2012).

Endolysins targeting Gram-positive bacteria are generally composed of multiple domains, typically one or more enzymatically active domains (EADs) at the N terminus and a CBD at the C terminus. On the other hand, endolysins targeting Gram-negative bacteria are usually small single-domain globular proteins that only consist of EADs (Broendum et al., 2018; Chang, 2020; Murray et al., 2021). However, some Gram-negative endolysins also contain a CBD at the N terminus, such as *Pseudomonas* endolysin KZ144 (Chang, 2020) and endolysin EL188 (Walmagh et al., 2012). EADs are responsible for the hydrolytic degradation of cell walls, while CBDs are responsible for the endolysin recognition and binding specificity toward the bacterial cell wall (Ajuebor et al., 2016; Broendum et al., 2018).

To date, several CBDs have been described, including the lysin motif domain (LysM), bacterial Src homology 3 domain (SH3b), choline-binding domain, and Cpl-7-like CBD which is also known as CW-7 repeats (Bustamante et al., 2017; Chang and Ryu, 2017; Mitkowski et al., 2019; Muraosa et al., 2019). SH3b domains are present and well conserved in many staphylococcal phage endolysins. It has also been shown to bind and recognize glycine cross-bridges for most staphylococci (Chang and Ryu, 2017; Mitkowski et al., 2019). LysM domains were first found in the C terminus of a bacteriophage lysozyme which binds to GlcNAc residues in the cell wall peptidoglycan but has since been described in various prokaryotic and eukaryotic proteins including both bacterial and fungal proteins (Muraosa et al., 2019). The choline-binding domain is mostly found in pneumococcal phages like Cpl-1 and Pal. It consists of six tandem repeats of approximately 20 amino acids that share high homology with the choline-binding motif, LytA (Hermoso et al., 2003). The Cpl-7-like CBD was named after the Cpl-7 endolysin, which contains a CBD that is made of three identical repeats of 42 amino acid residues. It recognizes the GlcNAc-MurNAc-L-Ala-D-isoGln muropeptide in the cell wall and can also hydrolyze pneumococcal cell walls that contain choline or ethanolamine (Bustamante et al., 2012, 2017). Unlike the choline-binding domain, which is exclusively found in pneumococcal proteins and pneumococcal phage proteins, the Cpl-7-like CBD is also found in phages infecting other species, like *S. agalactiae* and *S. suis* (Pritchard et al., 2007; Wang et al., 2009). Due to the CBD recognition and binding specificity, endolysins that target Gram-positive bacteria tend to have a narrow host range compared to Gram-negative lysins (Ajuebor et al., 2016; Broendum et al., 2018; Murray et al., 2021). Its target specificity range can vary from the entire bacterial genus to a specific bacterial strain, making it a potential antibiotic alternative (Broendum et al., 2018). Although some endolysins can also lyse across genus, these are not as common (Gilmer et al., 2013).

Endolysins can be further subclassified into five different subgroups depending on the cleavage site of the peptidoglycan cell wall by the EADs (Ajuebor et al., 2016; Chang, 2020; Zhou et al., 2020). These subgroups are (a) lysozyme (N-acetylmuramidases), (b) glycosidases (N-acetyl-β-D-glucosamidases), (c) amidases (N-acetylmuramoyl-L-alanine), (d) endopeptidases (L-alanoyl-D-glutamate), and (e) lytic transglycosylases (Ajuebor et al., 2016; Chang, 2020; Zhou et al., 2020; Murray et al., 2021).

Lytic transglycosylases are non-hydrolytic enzymes that cleave the glycosidic bond of the peptidoglycan cell wall to form 1,6-anhydro-N-acetylmuramic acid (1,6-anhydroMurNAc) and GlcNAc. On the other hand, lysozymes are hydrolytic enzymes that cleave the same glycosidic bond using a different cleavage mechanism to form MurNAc and GlcNAc (Dik et al., 2017; Broendum et al., 2018; Byun et al., 2018; Jenkins et al., 2019). Amidases, also known as peptidoglycan amidases, are involved in the cleavage of amide bonds between N-acetylmuramic acid and L-alanine, separating the glycan strand from the stem peptide (Ajuebor et al., 2016; Broendum et al., 2018; Vermassen et al., 2019). Glycosidases, also known as glycoside hydrolases, cleave the glycan bond at the reducing side of GlcNAc (Broendum et al., 2018; Vermassen et al., 2019). Last, endopeptidases target and cleave the dipeptide-binding motif consisting of L-lysine and D-alanine links (Vermassen et al., 2019; Murray et al., 2021).

## Sources and isolation of streptococcal endolysins

Streptococcal endolysins are encoded by streptococcal lytic or temperate phages, which exist naturally in the environment. Most reported streptococcal endolysins were either isolated and purified directly from the crude lysate of phage culture that shows lytic activity toward the host or from recombinant *Escherichia coli* that expresses cloned phage endolysins such as the PlyC endolysin. This endolysin was isolated and purified from the culture of phage C_1_, the first streptococcal *Podoviridae* isolated from a sewage plant (Nelson et al., 2001, 2003). PlyC exhibited lytic activity against groups A (*S. pyogenes*), C, and E *Streptococcus* with the strongest killing activity against group A *Streptococcus* (Nelson et al., 2001). The pneumococcal endolysin Pal was isolated from a CsCl density gradient centrifugation fraction of Dp-1 phage lysate that showed killing activity toward *S. pneumoniae* (Garcia, 1983). The Dp-1 phage was isolated from the throat of patients with upper respiratory disease similar to another pneumococcal phage Cp-1 (McDonnell et al., 1975; Ronda et al., 1981). Unlike Pal, the endolysin encoded by Cp-1 phage (Cpl-1) was isolated by cloning a DNA fragment of Cp-1, which showed similarity with the host autolysin LytA, into *E. coli* which produced a functional endolysin against *S. pneumoniae* (Garcia et al., 1987, 1988). *S. pneumoniae* is one of the major causes of pneumonia, meningitis, and sepsis in the respiratory tract, spinal fluid, and bloodstream, respectively. Therefore, the use of purified endolysins to treat pneumococcal infections is warranted due to an increasing trend of antimicrobial resistance (Jado et al., 2003; Hauser et al., 2012). Similarly, the recombinant endolysin gene from *S. suis* lytic phage SMP termed LySMP reportedly killed multiple strains of *S. suis*. The SMP phage was isolated from nasal swabs of Bama minipigs (Wang et al., 2009). Recently, two streptococcal endolysins 23TH_48 and SA01_53 were isolated using a similar approach, where the whole phage DNA of *S. infantis* 23TH siphovirus and *S. anginosus* SA01 siphovirus were sequenced, and the endolysin genes were cloned into *E. coli*. SA01_53 was found to lyse only its host target, while 23TH_48 exhibited a broader host range, which includes *S. pneumoniae*, thus making it a promising candidate to treat pneumococcal infections, in addition to Cpl-1 and Pal (van der Kamp et al., 2020).

**FIGURE 2:**
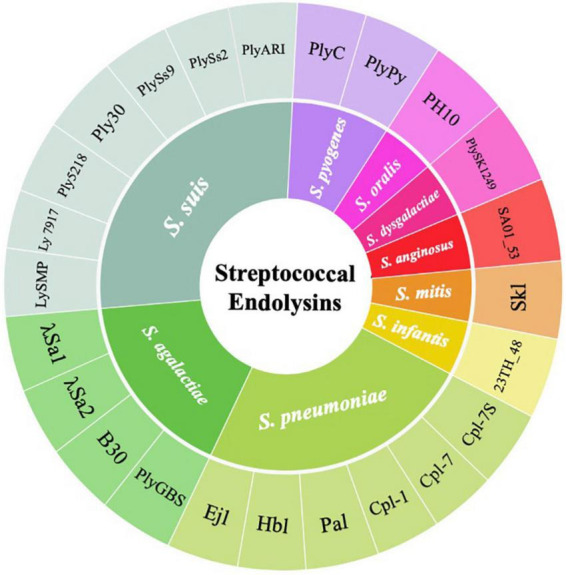
Wheel diagram summary depicting the classification of streptococcal endolysins reported to date and their corresponding streptococcal species targets.

Many other streptococcal endolysin genes were also identified and cloned from temperate streptococcal phages. Temperate phages can be isolated *via* induction with mitomycin C and other inducers, or discovered by bioinformatics analysis that predicts the presence of prophage in the host genome. For example, the pneumococcal endolysins Hbl and Ejl were isolated from temperate phages, *Siphoviridae* HB-3 and *Myoviridae* EJ-1, obtained from mitomycin C-induced pneumococcal cultures (Bernheimer, 1979; Garcia et al., 1997). Similarly, PlyGBS and B30 endolysins were isolated from GBS temperate phages from screening multiple GBS strains induced by mitomycin C (Pritchard et al., 2004; Cheng et al., 2005). Despite the wide use of mitomycin C to induce temperate phages into its lytic cycle, a huge number of streptococcal temperate phages were not able to turn on the lytic cycle when exposed to this compound (Domelier et al., 2009). Therefore, various streptococcal endolysins were identified by analyzing the presence of integrase and phage-related genes in the host genome. This indicated the presence of temperate phages, followed by subsequently elucidating its putative endolysins using bioinformatics approaches. Such candidate gene products were cloned into *E. coli* for expression and tested for their lytic activity *in vitro* and *in vivo*. This approach was used to obtain the PlyPy endolysin from *S. pyogenes*, PlySK1249 from *S. dysgalactiae*, λSa1 and λSa2 from *S. agalactiae*, Skl from *S. mitis*, and PlySs2 and PlySs9 from *S. suis* serotypes 2 and 9 (Nelson et al., 2001; Llull et al., 2006; Gilmer et al., 2013; Oechslin et al., 2013; Lood et al., 2014; Vander Elst et al., 2020). An overall classification of streptococcal endolysins reported to date and their corresponding streptococcal species targets are summarized in Figure 2.

## Enzymatically active domain and cell wall-binding domain of *Streptococci*-specific endolysins

Endolysins targeting *Streptococcus* species are unique due to the distinct types, numbers, and organization of EADs and CBDs (Table 1). These differences largely determine the strength and spectrum of the enzyme. Typically, the N-terminal domain consists of the EAD, and the C-terminal domain contains the CBD. However, some endolysins have an EAD at both N- and C-terminal domains with their CBD in the middle of the protein (Lood et al., 2014). Several endolysins hold more than one EAD, such as amidase, endopeptidase, and glycosidase domains, while others only have one EAD (Pritchard et al., 2007; McGowan et al., 2012; Vander Elst et al., 2020).

**TABLE 1:** Streptococcal phage lysin and their origin, host range, enzymatically active domains (EADs), and cell wall-binding domains (CBDs).

Phage origin	Endolysin	Host range	Enzymatically active domain	Cell wall-binding domain	References
*S. agalactiae*	B30	Group A, B, C, E, G streptococcus	*N-terminal* CHAP endopeptidase *Middle* Lysozyme	*C-terminal* Putative SH3b	Pritchard et al., 2004
	PlyGBS	GBS serotypes Ia, Ib, II, IIR, III, IIIR, V, GAS, GCS, GGS, GLS	*N-terminal* CHAP endopeptidase *Middle* Lysozyme	*C-terminal* Putative CBD	Cheng et al., 2005
	λSa1	*S. agalactiae*, *S. pneumoniae*, *S. aureus*	*N-terminal* Endopeptidase	*C-terminal* SH3b	Pritchard et al., 2007
	λSa2	*S. agalactiae*, *S. pneumoniae*, *S. aureus*	*N-terminal* Endopeptidase *C-terminal* Glycosidase	*Middle* Two copies of Cpl-7 CBD	Pritchard et al., 2007
*S. anginosus*	SA01_53	*S. anginosus*	*N-terminal* Lysozyme	*C-terminal* CW_7	van der Kamp et al., 2020
*S. dysgalactiae*	PlySK1249	*S. dysgalactiae, S. agalactiae*, *S pyogenes*	*N-terminal* Amidase-3 *C-terminal* CHAP endopeptidase	*Middle* LysM	Oechslin et al., 2013
*S. infantis*	23TH_48	*S. infantis*, *S. pneumoniae*	*N-terminal* Amidase-5	*C-terminal* Choline-binding domain	van der Kamp et al., 2020
*S. mitis*	Skl	*S. pneumoniae*, *S. mitis*	*N-terminal* CHAP amidase	*C-terminal* Choline-binding domain	Llull et al., 2006
*S. oralis*	PH10	*S. oralis, S. pneumoniae*, *S. mitis*	*N-terminal* Lysozyme	*C-terminal* Choline-binding domain	van der Ploeg, 2010
*S. pneumoniae*	Cpl-1 Cpl-7 Pal Hbl Ejl	*S. pneumoniae* *S. pneumoniae*	*N-terminal* Lysozyme *N-terminal* Amidase	*C-terminal* Choline-binding repeats (Cpl-1) CW_7 repeats (Cpl-7) *C-terminal* 6 Choline-binding repeats	Bustamante et al., 2010; Diez-Martinez et al., 2013; Garcia et al., 1998; Romero et al., 1990; Diaz et al., 1992; Sheehan et al., 1997
*S. pyogenes*	PlyC	Streptococcus groups A, C, E	*N-terminal PlyCA subunit* CHAP amidase Lysozyme	*C-terminal PlyCB subunits* An octamer of cell wall binding site	McGowan et al., 2012; Nelson et al., 2001; Nelson et al., 2006
	PlyPy	Group A, B, and C streptococcus, *S. uberis*	*N-terminal* CHAP endopeptidase	*C-terminal* SH3b	Lood et al., 2014
*S. suis*	LySMP	*S. suis* serotype 2, 7 and 9, *Streptococcus equi* ssp. *zooepidemicus*, *Staphylococcus aureus*	*N-terminal* Endopeptidase and glycosidase (sequence homology with λSA2 and 263V/R)	*C-terminal* Cpl-7 cell wall-binding domain	Wang et al., 2009
	Ly 7917	*S. suis, S. equi* ssp. *zooepidemicus*, *S. aureus*	*N-terminal* CHAP	*C-terminal* SH3b	Ji et al., 2015
	PlyARI	30 *S. suis* strains, *Staphylococcus aureus, and S. equi*, medium affinity to *S. pneumoniae* and *S. agalactiae*	*N-terminal* CHAP	*C-terminal* SH3b	Xiao et al., 2021
	PlySs2	MRSA, VISA, *S. suis*, *Listeria*, *Staphylococcus simulans*, *Staphylococcus epidermidis*, *S. equi*, *S. agalactiae*, *S. pyogenes*, *S. sanguinis*, GGS, GES, and *S. pneumoniae*	*N-terminal* CHAP	*C-terminal* SH3_5	Gilmer et al., 2013; Vander Elst et al., 2020
	PlySs9	*S. suis*, *S. uberis*	*N-terminal* Amidase *C-terminal* Endopeptidase	*Middle* LysM	Vander Elst et al., 2020
	Ply30	*S. suis*, *S. equi* subsp. *Zooepidemicus*	*N-terminal* CHAP	*Middle* SH3_5	Tang et al., 2015
	Ply5218	Multiple strains of *S. suis*	Unknown	Unknown	Zhang H. et al., 2016

CHAP, cysteine, histidine-dependent amidohydrolases/peptidases; GAS, group A Streptococcus; GBS, group B Streptococcus; GCS, group C Streptococcus; GES, Group E Streptococcus; GGS, group G Streptococcus; GLS, group L Streptococcus; MRSA, methicillin-resistant Staphylococcus aureus; VISA, vancomycin intermediate Staphylococcus aureus.

The domain organization of PlyC, which is made up of a multimeric holoenzyme consisting of two major subunits, PlyCA and PlyCB, makes it one of the most unique endolysins among other streptococcal endolysins (McGowan et al., 2012). It exhibits potent lytic activity against *S. pyogenes* (GAS) and can also kill other streptococcal species, including GCS, and group E *Streptococcus* (GES) (Nelson et al., 2001). The N-terminal subunit PlyCA is connected to the C-terminal subunit PlyCB that is made of an octamer arranged in a ring-like structure. PlyCA was found to carry two EADs, CHAP and lysozyme domains, which contribute to the potency of the endolysin. For the CBD, the octamer in the PlyCB subunit is predicted to have eight distinct binding sites, which may broaden the host range and/or share a similar binding site that could enhance cell wall binding (McGowan et al., 2012). The full capacity of lysis is only achieved with the complete holoenzyme structure containing PlyCA and PlyCB (Nelson et al., 2006). On another note, PlyPy is an endolysin targeting *S. pyogenes* with only one EAD (CHAP) and one CBD (SH3). It also displays a wide host range, including *S. pyogenes*, GBS, GCS, and *S. uberis*, where the killing activity was high, but it showed low or no killing activity on other species such as *S. suis*, *S. mutans*, *S. sobrinus*, *S. sanguinis*, and *S. pneumoniae* (Lood et al., 2014).

Most endolysins targeting *S. pneumoniae* possess only one EAD. Endolysins Cpl-1 and Cpl-7 were proposed to have lysozyme activity, while Pal, Hbl, and Ejl have amidase activity (Romero et al., 1990; Diaz et al., 1992; Sheehan et al., 1997; Garcia et al., 1998; Bustamante et al., 2010). Unlike the broad host range of *S. pyogenes* endolysins, *S. pneumoniae* endolysins can only lyse one species. This is because the CBD of these enzymes, except Cpl-7, shares a remarkable similarity with the choline-binding repeats of LytA, an autolysin of *S. pneumoniae* involved in cell division (Romero et al., 1990; Diaz et al., 1992; Sheehan et al., 1997; Garcia et al., 1998; Bustamante et al., 2010). The choline-binding motif is key to *S. pneumoniae* recognition, which possess choline-containing teichoic acids in the cell wall. Interestingly, the CBD of Cpl-7 consists of three tandem repeats of 42 amino acids, termed CW_7, that are unrelated to the choline-binding motif of other pneumococcal endolysins (Bustamante et al., 2010). This motif was also found in endolysins of *S. suis*, *S. anginosus*, and *S. agalactiae* phages (Pritchard et al., 2007; Wang et al., 2009). Later, a new Cpl-7 derivative named Cpl-7S was developed by substituting 15 amino acids that shifted the charge at neutral pH from negative to positive. It enhanced the activity against *S. pneumoniae* and extended its host range to other species like *S. pyogenes*, *E. faecalis*, and *S. mitis* (Diez-Martinez et al., 2013).

The endolysins of *S. suis* were shown to have more varieties in terms of its EAD and CBD than other streptococcal species. For example, PlySs2 contains only one EAD (CHAP) and one CBD (SH3_5), whereas PlySs9 contains two EADs, one amidase at the N terminus and one endopeptidase at the C terminus with a LysM CBD in the middle (Vander Elst et al., 2020). PlySs2 showed a wider host range than PlySs9, which included MRSA, VISA, *S. suis*, *Listeria*, *Staphylococcus simulans*, *Staphylococcus epidermidis*, *S. equi*, *S. agalactiae*, *S. pyogenes*, *S. sanguinis*, GGS, GES, and *S. pneumoniae*. By contrast, PlySs9 can only lyse *S. suis* and *S. uberis* (Gilmer et al., 2013; Vander Elst et al., 2020). Also, a *S. suis* endolysin called LySMP can lyse *S. suis* serotypes 2, 7, and 9; *Streptococcus equi* ssp. *Zooepidemicus*; and *S. aureus* (Wang et al., 2009). It also has two N-terminal EADs: endopeptidase and glycosidase as shown by sequence homology with λSA2 and 263V/R *S. agalactiae* prophage. The CBD of this enzyme shares a high similarity with the Cpl-7-like CBD of *S. pneumoniae* endolysin (Wang et al., 2009). Other *S. suis* endolysins Ply30, Ly7917, and PlyARI have one CHAP EAD and SH3 CBD. They were shown to kill multiple strains of *S. suis* and *S. equi* ssp. *zooepidemicus*, while PlyARI had medium affinity toward *S. pneumoniae* and *S. agalactiae* (Ji et al., 2015; Tang et al., 2015; Xiao et al., 2021).

*S. agalactiae* endolysins such as PlyGBS, B30, λSa1, and λSa2 were also shown to have different types of EAD and CBD. PlyGBS and B30 endolysins have similar CHAP domains at the N terminus and glycosidase domain at the middle of the enzyme as their EAD, but the C-terminal CBD of PlyGBS is unknown, while B30 has an SH3b CBD (Cheng et al., 2005; Donovan et al., 2006b). The host range exhibited by these endolysins were also different, whereby PlyGBS could kill multiple strains of GBS, whereas B30 has wider lytic activity including activity against groups A, B, C, E, and G *Streptococcus* (Pritchard et al., 2004; Cheng et al., 2005). In addition, λSa1 and λSa2 endolysins, which originated from the same host 263V/R *S. agalactiae*, were shown to share a similar endopeptidase domain at the N terminus, but λSa2 has an additional amidase domain at its C terminus (Pritchard et al., 2007). The CBDs of these enzymes were also different where λSa1 contains an SH3b domain at the C terminus, while λSa2 consists of two copies of Cpl-7 CBD at the middle of the protein (Pritchard et al., 2007). Nonetheless, both enzymes showed similar lytic activity on *S. agalactiae*, *S. pneumoniae*, and *S. aureus*.

In addition to the native endolysin, engineering the EAD or CBD by altering the amino acid sequence or combining the domains from different endolysins to create “chimeolysin” (Table 2) could improve the potency and/or the host range of the endolysin. For example, Cpl-7S is a derivative of Cpl-7 endolysin that contains 15 amino acid substitutions in the CW_7 repeats. This engineered endolysin was shown to kill multidrug-resistant strains of *S. pneumoniae*, *S. pyogenes*, *E. faecalis*, and *S. mitis*. (Diez-Martinez et al., 2013). The chimeolysin Cpl-711 is a modified version of Cpl-7, which contains the EAD (lysozyme) from Cpl-7, a linker from Cpl-1, and the choline-binding CBD from Cpl-1. It was shown to be more potent than their parental enzyme, having stronger bactericidal activity against multidrug-resistant *S. pneumoniae* and retaining a wide host range, reduced biofilm production, and increased protection against infections in mice by 50% (Diez-Martinez et al., 2015).

**TABLE 2:** List of engineered streptococcal endolysin (chimeolysin), including their origin, host range, enzymatically active domains (EADs), and cell wall-binding domains (CBDs).

Chimeolysin/Artilysin	Lysin origin	Host range	Enzymatically active domain	Cell-wall binding domain	References
B30 fused with lysostaphin	B30 endolysin and *Staphylococcus simulans* lysostaphin	*S. agalactiae*, *S. aureus*, *S. uberis*, *S. dysgalactiae*	(i) CHAP endopeptidase, lysozyme (from B30), and glycylglycine endopeptidase (from lysostaphin) (ii) CHAP endopeptidase (from B30) and glycylglycine endopeptidase (from lysostaphin)	(i) SH3b from B30 and SH3b from lysostaphin (ii) SH3b from lysostaphin only	Donovan et al., 2006a
ClyJ, ClyJ-3, ClyJ-3m	PlyC endolysin and gp20 putative lysin	*S. pneumoniae*	CHAP domain of PlyC and amidase-2 from gp20	Choline-binding domain from gp20	Luo et al., 2020; Yang et al., 2019
ClyR	PlyC endolysin and PlySs2 endolysin	*S. pyogenes*, *S. agalactiae*, *S. dysgalactiae*, *S. equi*, *S. mutans*, *S. pneumoniae*, *S. suis* and *S. uberis*, MRSA, VISA	CHAP domain of PlyC	SH3b domain from PlySs2 (PlySb)	Yang et al., 2015
Cpl-7S	Cpl-7 endolysin	*S. pneumoniae*, *S. pyogenes*, *E. faecalis*, and *S. mitis*	Lysozyme	15 amino acid substitution in CW_7 repeats	Diez-Martinez et al., 2013
Cpl-711	Cpl-1 endolysin and Cpl-7S endolysin	*S. pneumoniae*	Lysozyme from Cpl-7S	Linker and choline-binding domain from Cpl-1	Diez-Martinez et al., 2015
Csl2	Cpl-7 endolysin and LySMP endolysin	*S. suis*, *S. mitis*, *S. oralis*, *S. pseudopneumoniae*	Lysozyme from Cpl-7	Cpl-7-like CBD (CW_7 repeats) from LySMP	Vazquez et al., 2017
PL3	Pal endolysin and LytA autolysin	*S. pneumoniae* (including multi-resistant strains), *S. pseudopneumoniae*, *S. mitis*, *S. oralis*	Amidase from Pal	Choline-binding domain from LytA	Blazquez et al., 2016

CHAP, cysteine, histidine-dependent amidohydrolases/peptidases; MRSA, methicillin-resistant Staphylococcus aureus; VISA, vancomycin intermediate Staphylococcus aureus.

Furthermore, ClyR is another chimeolysin that contains the CHAP EAD from PlyC and the CBD from PlySs2 which consists of the SH3b domain (Yang et al., 2015). It was selected due to its robust activity and extended host range through a screening method using *E. coli*, which involved the shuffling of EAD and CBD from various endolysin donors to produce a library of random chimeras. The random combination of endolysins was co-transformed into *E. coli* that expresses ClyN, another endolysin that was able to lyse *E. coli* from within, thus allowing the tested chimeolysin to enter the extracellular environment. The resulting chimeolysins were screened by spotting the clones onto a layer of target bacterial species, where the formation of plaque indicated an active chimeolysin. This screening method voided the need of purifying the chimeolysins before screening. In addition to that, ClyJ is another chimeolysin that contains the CHAP domain from PlyC. The CBD of this enzyme was taken from a putative endolysin, gp20 of the streptococcal phage SPSL1, which contained a choline-binding domain similar to LytA (Yang et al., 2019). ClyJ exhibited stronger activity against *S. pneumoniae* than Cpl-1, and it produced no resistance in bacteria after prolonged exposure to the enzyme (Yang et al., 2019). ClyJ also has several derivatives denoted as ClyJ-3 and ClyJ-3m, which are essentially truncated versions of ClyJ (Luo et al., 2020). ClyJ-3 has a shorter linker than the parental ClyJ, whereas ClyJ-3m is a variant of ClyJ-3, which has a truncated C-terminal tail. Similar to ClyJ, ClyJ-3 forms a dimer conformation upon binding with a choline molecule, while ClyJ-3m forms a monomer upon choline binding. The bactericidal activity was greatly enhanced in ClyJ-3m when compared to the parental enzymes ClyJ-3 and ClyJ (Luo et al., 2020).

In another report, PL3 is a chimeolysin which was derived from pneumococcal endolysin Pal and pneumococcal autolysin LytA (Blazquez et al., 2016). The EAD was taken from the N-acetylmuramoyl-L-alanine amidase from Pal, whereas the CBD consisted of the choline-binding domain from LytA. PL3 not only showed stronger lytic activity against choline-containing species than the parental enzyme but also showed remarkable stability. This enzyme showed activity at low doses (0.5–5 μg/ml), and it was able to retain its enzymatic activity after 4 weeks of incubation at 37°C (Blazquez et al., 2016). In yet another report, Csl2 chimeolysin was a combination of the glycoside hydrolase EAD from the Cpl-7 endolysin and the Cpl-7-like CBD (CW_7) from LySMP (Vazquez et al., 2017). It showed efficient killing of *S. suis* and *S. mitis* groups and also reducing biofilm formation in *S. suis*. In addition to that, the lytic activity was also proven in an infected zebrafish model, where the bacterial load in the blood was significantly lower when treated with a high dosage of Csl2 (Vazquez et al., 2017).

B30 *S. agalactiae* endolysin has also been modified to include lysostaphin from *S. simulans* to produce a chimeolysin, and two chimeolysins were produced—one which consisted of the full-length B30 in combination with *S. simulans* lysostaphin and the other consisted of a C-terminally truncated B30 in combination with *S. simulans* (Donovan et al., 2006a). The first construct had CHAP and lysozyme domains from B30 and glycylglycine endopeptidase from lysostaphin as the EAD, and two SH3b domains—one from B30 and the other from lysostaphin. The second construct only had CHAP from B30 and the endopeptidase and SH3b from lysostaphin. Both constructs showed activity against *S. agalactiae*, *S. uberis*, *S. dysgalactiae*, and *S. aureus*. Also, these chimeolysins which were cloned in an eukaryotic vector were shown to be successfully expressed in mammalian cells, therefore providing the possibility of creating transgenic mice and cattle which are resistant to mastitis (Donovan et al., 2006a).

## Current applications of endolysins

### Animal farming and the dairy industry

The prevalence of AMR bacterial strains and the outbreaks in animal farming are major global issues that have increased significantly in recent years. Endolysin has been studied as an alternative bactericidal and preservative agent in the bid to alleviate food security issues. While many endolysins have been identified and studied extensively for their efficacy against specific pathogenic bacteria in the food industry (Ajuebor et al., 2016; Chang, 2020; Xu, 2021), streptococcal endolysins are more applicable in the control of diseases in animal farming and its products, especially milk affected by mastitic cattle.

According to Schmelcher and Loessner (2016), streptococcal phage λSA2 and B30 endolysin had a synergistic lytic effect against *S. agalactiae* and *S. uberis* in milk and in a mouse mastitis model. It was also reported that λSA2 lysin had stronger activity than B30 lysin, with a reduction of 3.5-log CFU/mL at 100 μg/mL. Streptococcal phage Ply700 endolysin was also found to have a rapid, calcium-dependent lysis against *S. uberis*, *S. pyogenes*, and *S. dysgalactiae* while exhibiting a slight lysis activity toward *S. agalactiae*. Studies also showed that Ply700 lysin has substantial killing activity (reduction of 81% bacterial count) in milk at 50 μg/mL (Celia et al., 2008). On the other hand, ClyR was reported to show strong lytic activity against *S. dysgalactiae* and *S. agalactiae* in pasteurized milk with a reduction of 5-log CFU/mL at 25 μg/mL (Yang et al., 2015).

Another endolysin, PlyC lysin was shown to eliminate more than 20 clinical isolates of *S. equi*, the causative agent of equine strangles, a highly contagious disease affecting horses which can lead to mortality (Hoopes et al., 2009). It was reported that 1 μg of PlyC lysin can sterilize 8-log CFU/mL of *S. equi* within 30 min, which is 1,000 times more active than a common disinfectant, Virkon-S, used in the control of livestock diseases. Similarly, LySMP, which has an extensive lytic spectrum, is expected to be able to control *S. suis* infection in swine, which causes arthritis, endocarditis, meningitis, pneumonia, and septicemia. Importantly, *S. suis* is a zoonotic agent with human outbreaks reported in China in 1998, 1999, and 2005; thus, the control of this disease is pertinent (Wang et al., 2009).

### Therapeutics

Phage therapy is an upcoming alternative therapeutic approach for a variety of animal and human infections by pathogenic bacteria. The lytic activity of various endolysins has also been tested and demonstrated by using *in vivo* mouse models. Cpl-1 lysin is a muramidase reported to rapidly lyse several other serotypes of *S. pneumoniae*. It has also been shown to eliminate *S. pneumoniae* nasopharyngeal colonization (Doehn et al., 2013). In addition, Cpl-1 dimers exhibited a 2-fold increase in antibacterial activity when compared to normal Cpl-1 (Resch et al., 2011). Ajuebor et al. (2016) also showed that Cpl-1 lysin could be aerosolized and administered through the respiratory airway to combat pneumococcal lung infections.

PlyC lysin, also referred to as the streptococcal C1 lysin, is a well-studied streptococcal endolysin with high specificity against group A, C, and E streptococci due to their two distinct EADs that have synergistic cleavage activities (Donovan and Foster-Frey, 2008; Shen et al., 2016; Vazquez et al., 2018; Linden et al., 2021). PlyC lysin was the first endolysin to exhibit *in vivo* efficacy following a nasopharyngeal challenge with *S. pyogenes* in mice. Other than that, PlyC lysin can also eliminate intracellular *S. pyogenes* by penetrating the epithelial cell lining of the respiratory tract (Vazquez et al., 2018; Linden et al., 2021).

Another endolysin, termed PlyPy lysin is derived from a prophage that infects *S. pyogenes* and was recently reported for its potential treatment of systemic bacteremia in mice. PlyPy lysin has a broad streptococci spectrum of activity with modest activity toward groups A, B, C, and E streptococci as well as *S. uberis* and *S. gordonii* (Xu, 2021). It is also the first *S. pyogenes* endolysin to be identified with a mechanism of action distinct from PlyC lysin (Donovan and Foster-Frey, 2008; Linden et al., 2021).

### Biofilm elimination

Many pathogenic bacteria produce biofilm, resulting in resistance to many antimicrobial treatments, which is a major concern in food and clinical settings (Ajuebor et al., 2016; Ur Rahman et al., 2021). Shen et al. (2016) showed that PlyC lysin could rapidly degrade and kill *S. pyogenes* within the biofilm matrixes. Cpl-711 lysin, a chimeolysin with the fusion of Cpl-7 and Cpl-1, has improved antibiofilm and antimicrobial activities *in vitro* and *in vivo* over either of the parental enzymes (Linden et al., 2021). Studies have shown that treatment with Cpl-711 strongly reduced the attachment of *S. pneumoniae* to human epithelial cells, and a single intranasal dose of Cpl-711 significantly reduced nasopharyngeal colonization in a mouse model (Vazquez et al., 2018).

LySMP lysin, found in *S. suis* serotype 2 bacteriophage, was tested for lytic activity against *S. suis* biofilm formation. LySMP lysin has been shown to eliminate more than 80% of biofilm when compared to phages or antibiotics alone (Meng et al., 2011). In addition to that, studies have shown that treatment using 50 μg/mL of ClyR lysin can reduce the biofilm of *S. mutans* and *S. sobrinus* within 5 min. It also shows that continuous administration of ClyR lysin in rat models infected with *S. mutans* and *S. sobrinus* can lower the severity for over 40 days (Xu et al., 2018).

## Advantages and limitations of endolysin therapy

Endolysins are highly specific and only lyse target bacteria depending on the presence of respective binding ligands with different cleavage mechanisms. This specificity has contributed to the narrow spectrum of endolysin lytic activity, which causes minimal disruption to other microbiota, thus giving it a huge advantage over broad range of antibiotics (Nayak et al., 2019; Schmelcher and Loessner, 2021). Studies have also shown that using more than one endolysin or combining endolysins with other antimicrobial agents enables a synergistic effect against bacterial infections. This can help reduce the required dose used for treatment and can also enhance the efficacy of the treatment (Nayak et al., 2019; Murray et al., 2021). For example, a study by Letrado et al. (2018) showed that the combination of lysin Cpl-711 with amoxicillin or cefotaxime resulted in a synergistic effect against MDR clinical isolates of *S. pneumoniae*.

The development of drug resistance in bacteria toward endolysin is also lower than that in antibiotics. Antibiotics work by interfering with the bacterial cell wall to prevent the growth and replication of bacteria, while endolysins work by lysing the bacterial cell wall. Furthermore, the binding and cleavage of endolysins target highly conserved regions of the cell wall, which are unlikely to mutate. Other than that, endolysins have not been reported to show any significant negative side effects or toxicity to mammalian cells (Romero-Calle et al., 2019). Endolysins also have a low environmental impact compared to antibiotics. For example, endolysins are composed mostly of proteins and can be rapidly inactivated or degraded by environmental factors, while non-metabolized antibiotics or residues that are released into the environment will have an impact on the environment that can indirectly accelerate the emergence of bacterial MDR (Loc-Carrillo and Abedon, 2011; Aslam et al., 2018; Alves-Barroco et al., 2020).

Although endolysins have many advantages, there are some challenges that limit the use of endolysin therapy. Endolysins show good efficacy in treating Gram-positive bacteria but are mostly ineffective against Gram-negative bacteria due to the outer membrane barrier. Fortunately, this problem can be potentially resolved with the addition of ethylenediaminetetraacetic acid (EDTA), which helps permeabilize the cell membrane. EDTA is a chelating agent that disrupts the outer membrane and unveils the peptidoglycan cell wall for lysis (Abdelrahman et al., 2021; Murray et al., 2021). In addition, artilyzation of Gram-negative endolysins can also help overcome this limitation. Furthermore, the encapsulation of endolysins into delivery vehicles such as chitosan nanoparticles and mucoadhesive film approaches can also be studied to enhance their bioavailability (Tan et al., 2019; Gondil and Chhibber, 2021).

To date, the usage of phage therapy in most countries still faces regulatory issues due to the lack of established clinical trials and safety data. However, phage therapy remains active in places such as Poland and Georgia, which provide personalized phage treatments for patients with antibiotic-resistant infections. The regulatory framework of established phage therapy is still on-going as there are little phage-related products licensed and marketed to date. Therefore, further evidence of successful human trials in phage therapy that can help current regulatory agencies gain practicable insights into phage therapy is needed (Fauconnier, 2019; Pires et al., 2020).

In the clinical setting, another crucial limitation of endolysins is the possibility of immune reactions as endolysins are ultimately proteins. The inflammatory responses and toxicity of endolysins have been tested and evaluated in animal models, and some endolysins do indeed provoke an immune response when they are used systematically (Abdelrahman et al., 2021). However, antibodies are weak inactivators of endolysins and were found to be unable to diminish the lytic capability of endolysins in the studies reported (Loeffler et al., 2003; Zhang L. et al., 2016). Nevertheless, the half-life of endolysins could be shortened due to host immune response as observed for the Cpl-1 endolysin in mice, which could indicate that multiple dosage may be required for eradication of infections (Loeffler et al., 2003). In addition, endolysins have also been found to be largely non-toxic to hosts (Murray et al., 2021). It should however be noted that in a study involving repeated dosage of endolysin treatment in canine with daily injection of the endolysin SAL200 against *Staphylococcus aureus* for more than 1 week, there were some transient abnormal clinical signs observed, which included subdued behavior and vomiting (Jun et al., 2014). While the symptoms were mild and self-limiting (resolved in 1 h), more clinical trials are needed to further accentuate the safety profile of endolysins for treatment.

Despite multiple studies highlighting the potential of endolysins as an antibiotic alternative to combat AMR, its current cost remains a stumbling block at this point in time in addition to the limitations highlighted before. As with all protein-based therapeutics, cost is usually a constraint when it comes to practical industrial adoption owing to relatively high production, purification, storage, and supply chain expenses relative to conventional synthetic-based equivalents (Love et al., 2018; Garcia et al., 2019; Gontijo et al., 2021; Lee et al., 2022). Hence, economic analyses and *in silico* model simulations such as response surface methodology for large-scale production and process conditions including media optimization and bioreactor conditions can help reduce costs (Krysiak-Baltyn et al., 2018; Pournejati and Karbalaei-Heidari, 2020). Purification costs should also be taken into consideration, especially purification from endotoxins, since *E. coli* is the most commonly used host for recombinant endolysins. Alternatively, Gram-positive microbial cell factories such as lactic acid bacteria can be used to produce endolysins. They are generally recognized as safe (GRAS), do not produce endotoxins, and can double up as a delivery vehicle, which may rid the need for purification, especially when the endolysin is secreted out of the cell (Chandran et al., 2022; Lee et al., 2022). Continuous investments are required for various sectors to embrace and adopt new techniques more quickly, while endorsement of phage and endolysin-based products by policymakers is pivotal when pushing them as mainstream antimicrobials.

## Conclusion

Phage therapy is currently a popular area of research with a high potential to become a treatment option for bacterial infections, including MDR bacteria. Their high recognition and binding specificity range can vary from the entire bacterial genus to a specific bacterial strain, making them a good candidate to replace antibiotic therapy. While some endolysins also can lyse across genus, these are not common. Further *in vivo* studies and clinical trials for various endolysins targeting different bacterial species are needed to ensure phage-derived lysins does not cause any form of toxicity, immunoreactions, and resistance to the target. Other than that, the development of chimeolysins and artilysins by using protein engineering and immune engineering has the potential to not only further improve lytic activity and target host range but also overcome undesired immunological responses when used systematically.

## Author contributions

KW and MM wrote the main manuscript text. KW, MM, and MT prepared figures and tables. All authors read and approved the final manuscript.
